# 3,5-Dibromo-2,2,6,6,7,7-hexa­methyl­octane-4-one

**DOI:** 10.1107/S160053681204603X

**Published:** 2012-11-17

**Authors:** Ted S. Sorensen, Jianjun Hou, Masood Parvez

**Affiliations:** a Department of Chemistry, The University of Calgary, 2500 University Drive NW, Calgary, Alberta, Canada T2N 1N4

## Abstract

In the title mol­ecule, C_14_H_26_Br_2_O, the central carbonyl group (C_3_O) is essentially planar (r.m.s. deviation = 0.0021 Å). The Br atoms lie on the same side of the mol­ecule and are approximately *syn*, with a Br—C⋯C—Br torsion angle of −43.52 (13)°. The crystal structure is devoid of any specific inter­molecular inter­actions.

## Related literature

For background literature and the synthesis and crystal structures of related compounds, see: Parvez *et al.* (2002 ▶)
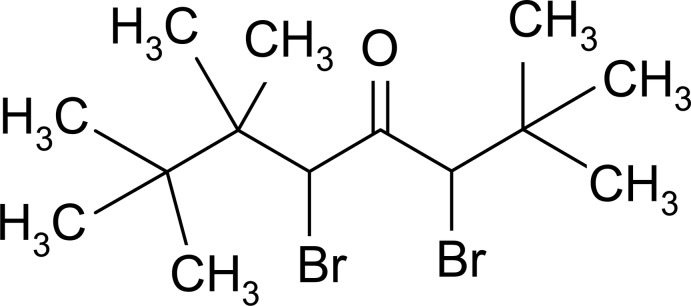


## Experimental

### 

#### Crystal data

C_14_H_26_Br_2_O
*M* *_r_* = 370.17
Monoclinic, 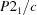

*a* = 14.602 (5) Å
*b* = 9.963 (2) Å
*c* = 10.974 (4) Å
β = 93.321 (13)°
*V* = 1593.8 (9) Å^3^
*Z* = 4
Mo *K*α radiation
μ = 5.07 mm^−1^
*T* = 123 K
0.16 × 0.14 × 0.04 mm

#### Data collection

Nonius APEXII CCD diffractometer
Absorption correction: multi-scan (*SORTAV*; Blessing, 1997 ▶) *T*_min_ = 0.498, *T*_max_ = 0.823
6698 measured reflections
3630 independent reflections
3044 reflections with *I* > 2σ(*I*)
*R*_int_ = 0.022

#### Refinement

*R*[*F* ^2^ > 2σ(*F* ^2^)] = 0.029
*wR*(*F* ^2^) = 0.073
*S* = 1.06
3630 reflections
162 parameters
H-atom parameters constrained
Δρ_max_ = 0.49 e Å^−3^
Δρ_min_ = −0.62 e Å^−3^

### 

Data collection: *COLLECT* (Hooft, 1998 ▶); cell refinement: *DENZO* (Otwinowski & Minor, 1997 ▶); data reduction: *SCALEPACK* (Otwinowski & Minor, 1997 ▶); program(s) used to solve structure: *SHELXS97* (Sheldrick, 2008 ▶); program(s) used to refine structure: *SHELXL97* (Sheldrick, 2008 ▶); molecular graphics: *ORTEP-3 for Windows* (Farrugia, 1997 ▶); software used to prepare material for publication: *SHELXL97*.

## Supplementary Material

Crystal structure: contains datablock(s) global, I. DOI: 10.1107/S160053681204603X/tk5169sup1.cif

Structure factors: contains datablock(s) I. DOI: 10.1107/S160053681204603X/tk5169Isup2.hkl

Supplementary material file. DOI: 10.1107/S160053681204603X/tk5169Isup3.cml

Additional supplementary materials:  crystallographic information; 3D view; checkCIF report

## Figures and Tables

**Fig. 1.**
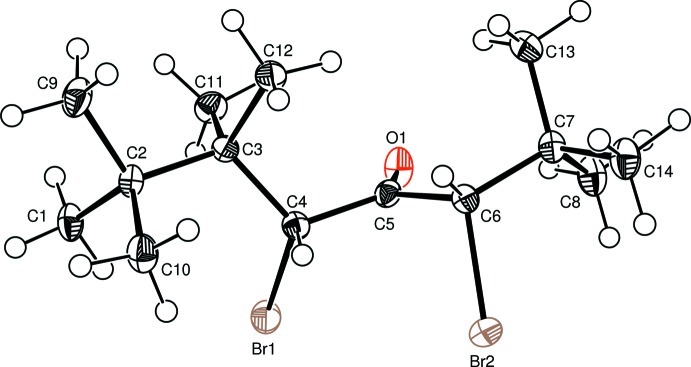
ORTEP drawing (Farrugia, 1997) of the title molecule with the displacement ellipsoids plotted at 50% probability level; H atoms are presented as small spheres of arbitrary radius.

**Fig. 2.**
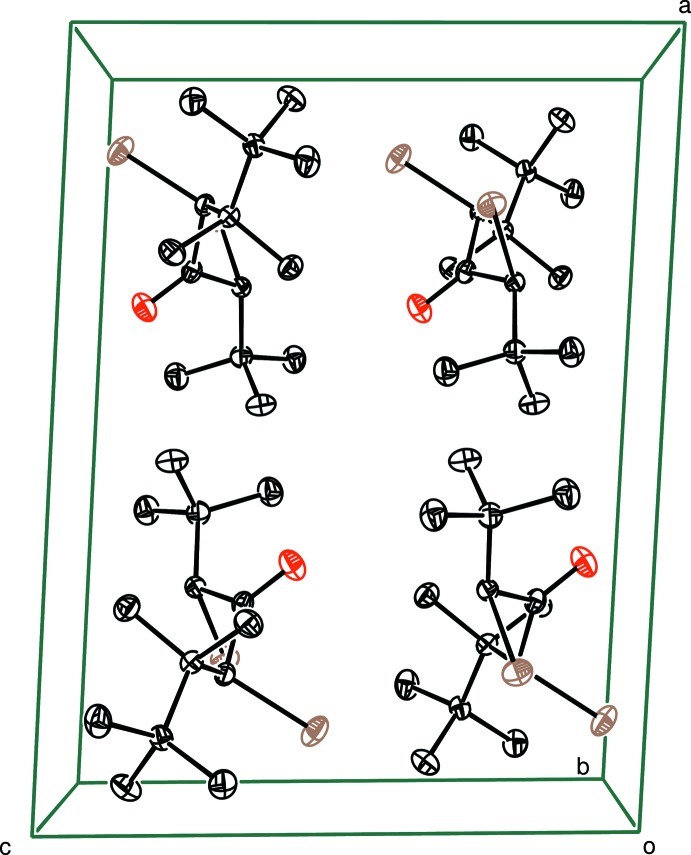
A view of the unit cell packing of the crystal structure of the title compound. H atoms were omitted for clarity.

## supplementary crystallographic information

### Comment

In continuation of our investigations on the characterization of ketones, we now report the crystal structure of the title compound (Fig. 1). The molecular dimensions in the title molecule agree very well with the corresponding molecular dimensions reported in closely related compounds (Parvez *et al.*, 2002). The crystal structure (Fig. 2) is devoid of any intermolecular interactions.

### Experimental

The synthesis of the title compound and related compounds has been reported earlier (Parvez *et al.*, 2002). Crystals suitable for crystallographic studies were grown from pentane/CH_2_Cl_2_ (1:1).

### Refinement

Though the H-atoms were observable in the difference electron density maps they were included at geometrically idealized positions with C—H distances = 1.00 and 0.98 Å for methine and methyl type H-atoms, respectively. The H-atoms were assigned *U*_iso_ = 1.2 and 1.5 times *U*_eq_(methine and methyl C-atoms, respectively).

### Crystal data

**Table:**

C_14_H_26_Br_2_O	*F*(000) = 752
*M**_r_* = 370.17	*D*_x_ = 1.543 Mg m^−^^3^
Monoclinic, *P*2_1_/*c*	Mo *K*α radiation, λ = 0.71073 Å
Hall symbol: -P 2ybc	Cell parameters from 6698 reflections
*a* = 14.602 (5) Å	θ = 3.1–27.5°
*b* = 9.963 (2) Å	µ = 5.07 mm^−^^1^
*c* = 10.974 (4) Å	*T* = 123 K
β = 93.321 (13)°	Plate, colourless
*V* = 1593.8 (9) Å^3^	0.16 × 0.14 × 0.04 mm
*Z* = 4	

### Data collection

**Table:**

Nonius APEXII CCD diffractometer	3630 independent reflections
Radiation source: fine-focus sealed tube	3044 reflections with *I* > 2σ(*I*)
Graphite monochromator	*R*_int_ = 0.022
ω and φ scans	θ_max_ = 27.5°, θ_min_ = 3.2°
Absorption correction: multi-scan (*SORTAV*; Blessing, 1997)	*h* = −18→18
*T*_min_ = 0.498, *T*_max_ = 0.823	*k* = −12→12
6698 measured reflections	*l* = −14→14

### Refinement

**Table:**

Refinement on *F*^2^	Primary atom site location: structure-invariant direct methods
Least-squares matrix: full	Secondary atom site location: difference Fourier map
*R*[*F*^2^ > 2σ(*F*^2^)] = 0.029	Hydrogen site location: inferred from neighbouring sites
*wR*(*F*^2^) = 0.073	H-atom parameters constrained
*S* = 1.06	*w* = 1/[σ^2^(*F*_o_^2^) + (0.0402*P*)^2^ + 0.279*P*] where *P* = (*F*_o_^2^ + 2*F*_c_^2^)/3
3630 reflections	(Δ/σ)_max_ < 0.001
162 parameters	Δρ_max_ = 0.49 e Å^−^^3^
0 restraints	Δρ_min_ = −0.62 e Å^−^^3^

### Special details

**Table:**

Geometry. All e.s.d.'s (except the e.s.d. in the dihedral angle between two l.s. planes) are estimated using the full covariance matrix. The cell e.s.d.'s are taken into account individually in the estimation of e.s.d.'s in distances, angles and torsion angles; correlations between e.s.d.'s in cell parameters are only used when they are defined by crystal symmetry. An approximate (isotropic) treatment of cell e.s.d.'s is used for estimating e.s.d.'s involving l.s. planes.
Refinement. Refinement of *F*^2^ against ALL reflections. The weighted *R*-factor *wR* and goodness of fit *S* are based on *F*^2^, conventional *R*-factors *R* are based on *F*, with *F* set to zero for negative *F*^2^. The threshold expression of *F*^2^ > σ(*F*^2^) is used only for calculating *R*-factors(gt) *etc*. and is not relevant to the choice of reflections for refinement. *R*-factors based on *F*^2^ are statistically about twice as large as those based on *F*, and *R*- factors based on ALL data will be even larger.

### Fractional atomic coordinates and isotropic or equivalent isotropic displacement parameters (Å^2^)

**Table:**

	*x*	*y*	*z*	*U*_iso_*/*U*_eq_	
Br1	0.119984 (18)	0.34952 (2)	0.04616 (2)	0.02780 (9)	
Br2	0.198646 (17)	0.02636 (2)	0.21493 (2)	0.02471 (9)	
O1	0.32951 (12)	0.29847 (16)	0.09933 (14)	0.0265 (4)	
C1	0.07327 (18)	0.6488 (2)	0.1864 (2)	0.0244 (5)	
H1A	0.0159	0.6851	0.2148	0.037*	
H1B	0.0595	0.5852	0.1197	0.037*	
H1C	0.1108	0.7223	0.1572	0.037*	
C2	0.12605 (16)	0.5761 (2)	0.29239 (18)	0.0177 (4)	
C3	0.21494 (16)	0.5004 (2)	0.25226 (19)	0.0170 (4)	
C4	0.19353 (16)	0.3561 (2)	0.20209 (18)	0.0171 (5)	
H4	0.1584	0.3079	0.2642	0.021*	
C5	0.27965 (16)	0.2724 (2)	0.18064 (18)	0.0175 (4)	
C6	0.29800 (15)	0.1531 (2)	0.26737 (19)	0.0168 (4)	
H6	0.2863	0.1840	0.3518	0.020*	
C7	0.39337 (16)	0.0872 (2)	0.27212 (19)	0.0201 (5)	
C8	0.42040 (19)	0.0297 (2)	0.1499 (2)	0.0276 (6)	
H8A	0.4825	−0.0078	0.1593	0.041*	
H8B	0.4189	0.1012	0.0885	0.041*	
H8C	0.3771	−0.0412	0.1236	0.041*	
C9	0.15287 (19)	0.6853 (2)	0.3883 (2)	0.0266 (5)	
H9A	0.1732	0.6423	0.4656	0.040*	
H9B	0.0996	0.7424	0.4010	0.040*	
H9C	0.2028	0.7402	0.3591	0.040*	
C10	0.05875 (18)	0.4806 (2)	0.3510 (2)	0.0249 (5)	
H10A	0.0916	0.4279	0.4150	0.037*	
H10B	0.0314	0.4200	0.2887	0.037*	
H10C	0.0102	0.5329	0.3869	0.037*	
C11	0.26542 (17)	0.5849 (2)	0.1600 (2)	0.0242 (5)	
H11A	0.3257	0.5451	0.1483	0.036*	
H11B	0.2734	0.6766	0.1910	0.036*	
H11C	0.2294	0.5868	0.0818	0.036*	
C12	0.28176 (18)	0.4758 (2)	0.3644 (2)	0.0227 (5)	
H12A	0.3337	0.4217	0.3401	0.034*	
H12B	0.2497	0.4281	0.4274	0.034*	
H12C	0.3043	0.5621	0.3969	0.034*	
C13	0.46246 (18)	0.1962 (2)	0.3165 (2)	0.0278 (5)	
H13A	0.5239	0.1569	0.3269	0.042*	
H13B	0.4446	0.2324	0.3947	0.042*	
H13C	0.4627	0.2686	0.2561	0.042*	
C14	0.39600 (19)	−0.0260 (2)	0.3682 (2)	0.0267 (5)	
H14A	0.4584	−0.0621	0.3785	0.040*	
H14B	0.3537	−0.0977	0.3409	0.040*	
H14C	0.3776	0.0099	0.4463	0.040*	

### Atomic displacement parameters (Å^2^)

**Table:**

	*U*^11^	*U*^22^	*U*^33^	*U*^12^	*U*^13^	*U*^23^
Br1	0.03166 (17)	0.02673 (14)	0.02357 (13)	0.00707 (10)	−0.01094 (10)	−0.00763 (9)
Br2	0.02415 (15)	0.01923 (13)	0.03042 (14)	−0.00386 (9)	−0.00116 (10)	−0.00072 (8)
O1	0.0318 (11)	0.0245 (8)	0.0242 (8)	0.0076 (7)	0.0108 (7)	0.0059 (6)
C1	0.0284 (15)	0.0209 (11)	0.0237 (11)	0.0097 (9)	−0.0002 (10)	0.0027 (8)
C2	0.0207 (13)	0.0153 (10)	0.0173 (10)	0.0038 (9)	0.0027 (9)	0.0006 (8)
C3	0.0171 (12)	0.0170 (10)	0.0166 (10)	0.0004 (9)	−0.0012 (9)	−0.0004 (8)
C4	0.0176 (12)	0.0196 (11)	0.0139 (10)	0.0016 (8)	−0.0008 (9)	−0.0009 (8)
C5	0.0212 (12)	0.0155 (10)	0.0156 (10)	0.0008 (9)	−0.0011 (9)	−0.0022 (8)
C6	0.0165 (12)	0.0183 (11)	0.0157 (10)	0.0015 (8)	0.0016 (8)	0.0003 (7)
C7	0.0213 (13)	0.0191 (11)	0.0197 (10)	0.0036 (9)	0.0010 (9)	0.0004 (8)
C8	0.0312 (15)	0.0285 (13)	0.0234 (12)	0.0100 (11)	0.0048 (10)	0.0019 (9)
C9	0.0354 (16)	0.0222 (12)	0.0221 (11)	0.0069 (10)	0.0016 (10)	−0.0052 (9)
C10	0.0238 (14)	0.0220 (11)	0.0300 (12)	0.0027 (10)	0.0097 (10)	0.0047 (9)
C11	0.0244 (14)	0.0227 (12)	0.0260 (12)	−0.0034 (10)	0.0053 (10)	0.0014 (9)
C12	0.0227 (13)	0.0232 (12)	0.0216 (11)	0.0023 (9)	−0.0035 (9)	−0.0042 (8)
C13	0.0182 (13)	0.0286 (13)	0.0362 (13)	0.0009 (10)	−0.0017 (11)	0.0003 (10)
C14	0.0327 (16)	0.0246 (12)	0.0229 (12)	0.0075 (10)	0.0020 (10)	0.0042 (9)

### Geometric parameters (Å, º)

**Table:**

Br1—C4	1.968 (2)	C8—H8A	0.9800
Br2—C6	1.984 (2)	C8—H8B	0.9800
O1—C5	1.212 (3)	C8—H8C	0.9800
C1—C2	1.539 (3)	C9—H9A	0.9800
C1—H1A	0.9800	C9—H9B	0.9800
C1—H1B	0.9800	C9—H9C	0.9800
C1—H1C	0.9800	C10—H10A	0.9800
C2—C10	1.536 (3)	C10—H10B	0.9800
C2—C9	1.548 (3)	C10—H10C	0.9800
C2—C3	1.585 (3)	C11—H11A	0.9800
C3—C11	1.537 (3)	C11—H11B	0.9800
C3—C12	1.545 (3)	C11—H11C	0.9800
C3—C4	1.565 (3)	C12—H12A	0.9800
C4—C5	1.538 (3)	C12—H12B	0.9800
C4—H4	1.0000	C12—H12C	0.9800
C5—C6	1.536 (3)	C13—H13A	0.9800
C6—C7	1.538 (3)	C13—H13B	0.9800
C6—H6	1.0000	C13—H13C	0.9800
C7—C8	1.531 (3)	C14—H14A	0.9800
C7—C13	1.542 (3)	C14—H14B	0.9800
C7—C14	1.543 (3)	C14—H14C	0.9800
			
C2—C1—H1A	109.5	H8A—C8—H8B	109.5
C2—C1—H1B	109.5	C7—C8—H8C	109.5
H1A—C1—H1B	109.5	H8A—C8—H8C	109.5
C2—C1—H1C	109.5	H8B—C8—H8C	109.5
H1A—C1—H1C	109.5	C2—C9—H9A	109.5
H1B—C1—H1C	109.5	C2—C9—H9B	109.5
C10—C2—C1	107.7 (2)	H9A—C9—H9B	109.5
C10—C2—C9	107.05 (18)	C2—C9—H9C	109.5
C1—C2—C9	106.23 (18)	H9A—C9—H9C	109.5
C10—C2—C3	112.07 (17)	H9B—C9—H9C	109.5
C1—C2—C3	113.33 (17)	C2—C10—H10A	109.5
C9—C2—C3	110.13 (19)	C2—C10—H10B	109.5
C11—C3—C12	107.85 (19)	H10A—C10—H10B	109.5
C11—C3—C4	111.40 (17)	C2—C10—H10C	109.5
C12—C3—C4	103.79 (17)	H10A—C10—H10C	109.5
C11—C3—C2	110.76 (18)	H10B—C10—H10C	109.5
C12—C3—C2	110.09 (17)	C3—C11—H11A	109.5
C4—C3—C2	112.63 (18)	C3—C11—H11B	109.5
C5—C4—C3	113.79 (18)	H11A—C11—H11B	109.5
C5—C4—Br1	105.00 (13)	C3—C11—H11C	109.5
C3—C4—Br1	115.10 (14)	H11A—C11—H11C	109.5
C5—C4—H4	107.5	H11B—C11—H11C	109.5
C3—C4—H4	107.5	C3—C12—H12A	109.5
Br1—C4—H4	107.5	C3—C12—H12B	109.5
O1—C5—C6	122.1 (2)	H12A—C12—H12B	109.5
O1—C5—C4	121.87 (19)	C3—C12—H12C	109.5
C6—C5—C4	116.05 (17)	H12A—C12—H12C	109.5
C5—C6—C7	118.46 (18)	H12B—C12—H12C	109.5
C5—C6—Br2	102.39 (14)	C7—C13—H13A	109.5
C7—C6—Br2	112.55 (14)	C7—C13—H13B	109.5
C5—C6—H6	107.6	H13A—C13—H13B	109.5
C7—C6—H6	107.6	C7—C13—H13C	109.5
Br2—C6—H6	107.6	H13A—C13—H13C	109.5
C8—C7—C6	114.14 (19)	H13B—C13—H13C	109.5
C8—C7—C13	110.1 (2)	C7—C14—H14A	109.5
C6—C7—C13	106.53 (18)	C7—C14—H14B	109.5
C8—C7—C14	109.12 (18)	H14A—C14—H14B	109.5
C6—C7—C14	108.79 (19)	C7—C14—H14C	109.5
C13—C7—C14	108.0 (2)	H14A—C14—H14C	109.5
C7—C8—H8A	109.5	H14B—C14—H14C	109.5
C7—C8—H8B	109.5		
			
C10—C2—C3—C11	162.2 (2)	C3—C4—C5—O1	−69.9 (3)
C1—C2—C3—C11	40.0 (3)	Br1—C4—C5—O1	56.8 (2)
C9—C2—C3—C11	−78.8 (2)	C3—C4—C5—C6	110.8 (2)
C10—C2—C3—C12	−78.7 (2)	Br1—C4—C5—C6	−122.49 (16)
C1—C2—C3—C12	159.22 (19)	O1—C5—C6—C7	15.5 (3)
C9—C2—C3—C12	40.4 (2)	C4—C5—C6—C7	−165.23 (18)
C10—C2—C3—C4	36.7 (2)	O1—C5—C6—Br2	−109.0 (2)
C1—C2—C3—C4	−85.5 (2)	C4—C5—C6—Br2	70.32 (19)
C9—C2—C3—C4	155.70 (17)	C5—C6—C7—C8	−59.8 (3)
C11—C3—C4—C5	63.6 (2)	Br2—C6—C7—C8	59.5 (2)
C12—C3—C4—C5	−52.2 (2)	C5—C6—C7—C13	61.9 (2)
C2—C3—C4—C5	−171.27 (17)	Br2—C6—C7—C13	−178.84 (14)
C11—C3—C4—Br1	−57.7 (2)	C5—C6—C7—C14	178.07 (18)
C12—C3—C4—Br1	−173.47 (14)	Br2—C6—C7—C14	−62.6 (2)
C2—C3—C4—Br1	67.5 (2)		
